# Prognostic Role of MMP2, MMP9, and IL-1β Markers in Cardiac Allograft Rejection After Transplantation

**DOI:** 10.3390/ijms26189136

**Published:** 2025-09-18

**Authors:** Gabriela Patrichi, Catalin-Bogdan Satala, Andrei Ionut Patrichi, Alexandru-Nicusor Tomut, Ovidiu Simion Cotoi, Horatiu Suciu, Anca Ileana Sin

**Affiliations:** 1 Molecular and Cell Biology Department, George Emil Palade University of Medicine, Pharmacy, Sciences, and Technology of Targu Mures, 540142 Targu Mures, Romania; gabriela.constantin@umfst.ro (G.P.); ileana.sin@umfst.ro (A.I.S.); 2 Pathology Department, Clinical Emergency County Hospital Targu Mures, 540136 Targu Mures, Romania; 3 Pathology Department, Faculty of Medicine and Pharmacy, Dunarea de Jos University of Galati, 800008 Galati, Romania; 4 Pathology Department, Clinical Emergency County Hospital Braila, 810325 Braila, Romania; 5 Pathology Department, George Emil Palade University of Medicine, Pharmacy, Sciences, and Technology of Targu Mures, 540142 Targu Mures, Romania; 6 Faculty of Medicine, George Emil Palade University of Medicine, Pharmacy, Sciences, and Technology of Targu Mures, 540142 Targu Mures, Romania; nicusortomut19@gmail.com; 7 Pathology Department, Mures Clinical County Hospital, 540011 Targu Mures, Romania; ovidiu.cotoi@umfst.ro; 8 Pathophysiology Department, George Emil Palade University of Medicine, Pharmacy, Sciences and Technology of Targu Mures, 540142 Targu Mures, Romania; 9 Emergency Institute for Cardiovascular Diseases and Transplant, 540136 Targu Mures, Romania; horatiu.suciu@umfst.ro; 10 Department of Surgery, George Emil Palade University of Medicine, Pharmacy, Sciences and Technology of Targu Mures, 540142 Targu Mures, Romania

**Keywords:** cardiac allograft rejection, immunohistochemistry, IL-1β, matrix metalloproteinases (MMP2, MMP9)

## Abstract

Cardiac allograft rejection remains a major cause of graft dysfunction post-transplant. While histology is the current diagnostic standard, it may miss early immune and inflammatory events. This study evaluated the immunohistochemical expression of matrix metalloproteinases 2 (MMP2), 9 (MMP9), and interleukin-1 beta (IL-1β) in cardiac transplant patients, correlating their expression with acute cellular rejection (ACR), antibody-mediated rejection (AMR), inflammation, vasculitis, the Quilty effect, and immune markers. Fifty-nine endomyocardial biopsy specimens were retrospectively analyzed. Immunohistochemical staining for MMP2, MMP9, and IL-1β was assessed based on nuclear, cytoplasmic, and membranous expression. Correlations were evaluated using Fisher’s exact test and odds ratios (ORs) with 95% confidence intervals (CIs). IL-1β nuclear expression showed strong associations with ACR (*p* = 0.0001), inflammation, vasculitis, and immune/endothelial markers (all *p* < 0.003). Nuclear MMP9 expression correlated with ACR and immune cell markers and was borderline significant for AMR (*p* ≈ 0.05). Cytoplasmic MMP2 (>50%) was significantly associated with AMR (OR = 7.47, *p* = 0.0002). No marker correlated with the Quilty effect. The immunohistochemical profiles of IL-1β and MMP9 support their involvement in immune-mediated injury in cardiac allograft rejection, with IL-1β emerging as a sensitive marker of early inflammation. MMP2 appears to be more relevant to humoral rejection processes. These findings suggest that selected tissue biomarkers may enhance diagnostic precision and support early detection of graft injury when integrated with conventional histology.

## 1. Introduction

Cardiac transplantation is currently the elective treatment for end-stage heart failure and, along with new immunosuppressive therapies, leads to better preservation of the heart graft and a lower rate of cardiac rejection, the immediate post-transplant complication [1]. Despite these improvements, the risk of cardiac rejection in the early postoperative period remains, so endomyocardial biopsy remains the gold standard in the routine surveillance and follow-up of transplant patients [2,3].

In 1990, the International Society for Heart and Lung Transplantation (ISHLT) proposed a scaled system to quantify acute cellular rejection (ACR) based on morphologic aspects of the cardiac graft, which was revised in 2004. It has served as a guideline in clinical therapeutic practice, with the later addition of pathologic diagnosis of antibody-mediated rejection (pAMR), as the role of these parameters in mediating cardiac allograft dysfunction is well established [4,5].

Generally, cardiac allograft rejection is morphologically characterized by the destruction of cardiac myocytes, inflammation, and severe fibrosis processes, leading to ventricular dysfunction of the transplanted heart [6,7]. Over time, a spectrum of immunologic changes has been reported as antibody-mediated rejection (AMR), which initially follows humoral rejection due to vasculitis and edema before the emergence of antibodies corresponding to these changes (anti-CD68 molecule [CD68], anti-platelet and endothelial cell adhesion molecule 1 [PECAM1/CD31], and anti-CD4 molecule [CD4]) and seric donor-specific antibodies (DSA) [8,9].

Ultimately, in 2006, the ISHLT Immunopathology Task Force defined the minimum criteria for immunopathologic evidence of antibody-mediated lesions, which included the presence of acute capillary vascular lesions and evidence of serologic levels of circulating antibodies [10]. Among these histopathologic changes were additional immunopathologic features, including immunoglobulin deposits, complement components, and post-transplant antibodies (anti-human leukocyte antigen [HLA] and non-HLA antibodies) [11,12,13]. These AMR changes are associated with significantly more severe cardiac graft dysfunction. Therefore, these mechanisms and circulating antibodies must be further investigated to facilitate future progress in treatment.

The extracellular matrix (ECM) components present in the cardiac interstitium provide cellular support and integrate extracellular signals and cellular responses. They also participate in inflammation regulation, as well as cardiac remodeling and function [14]. The balance between the synthesis and degradation of the ECM maintains the structural integrity of the myocardium. The ECM is primarily degraded by proteolytic enzymes known as matrix metalloproteinases (MMPs), with the most common subtypes found in the heart being MMP2 and MMP9 [15,16]. Recent research has increasingly highlighted the role of proinflammatory mediators such as IL-1β in driving vascular inflammation and endothelial activation, with emerging evidence detailing IL-1β secretion mechanisms and its downstream vascular effects [17]. Likewise, MMPs have been identified as key effectors in infection-related and inflammatory pathologies, contributing to extracellular matrix disruption, immune cell infiltration, and vascular barrier compromise—findings that underscore their dual role as potential biomarkers and therapeutic targets [18]. Taken together, these insights reinforce the rationale for investigating IL-1β and MMP expression in cardiac allograft rejection. Given the importance of maintaining the integrity of the cardiac ECM for the proper functioning of the cardiac allograft and the paucity of studies examining it, it is imperative to examine these proteases, which may offer new therapeutic strategies and perspectives for future approaches to cardiac transplantation pathology.

Interleukin 1 beta (IL1B/IL-1β) is a proinflammatory cytokine with a well-documented role in mediating ischemia–reperfusion injury and graft inflammation after cardiac transplantation via the transmembrane interleukin 1 receptor type 1 (IL1R1) [19,20,21]. Ischemia–reperfusion injury is a trigger for cardiac graft inflammation, which can result in allograft rejection. During these changes, oxygen deprivation leads to cellular destruction and the activation of apoptotic signaling cascades, including the proinflammatory cytokine IL-1β. Elevated levels of IL-1β have been associated with the activation of alloreactive T cells, one of the early changes in cardiac graft rejection [22,23,24]. Moreover, various studies using mouse cell cultures have demonstrated that IL-1β induces cardiac myocyte hypertrophy and reinitiates myocyte DNA synthesis [25].

## 2. Results

### 2.1. Clinical and Pathological Characteristics of the Selected Cases

In the study cohort (N = 59), the mean age was 56 years (SD ± 15.8), and most cases were male and over 40 years of age. In addition, most showed no histopathological changes compatible with ACR (81.6%). Of the remainder, 13.6% exhibited mild ACR (ISHLT grade 1R), characterized by interstitial or perivascular inflammatory infiltrates with up to one focus of myocyte damage; 3.4% exhibited moderate ACR (ISHLT grade 2R); and only 1.7% exhibited severe ACR (ISHLT grade 3R). Consistent with the current definitions, the criteria used to assess the grade of ACR included cardiomyocyte injury, inflammation, and histological changes suggestive of vasculitis.

**Figure 1:**
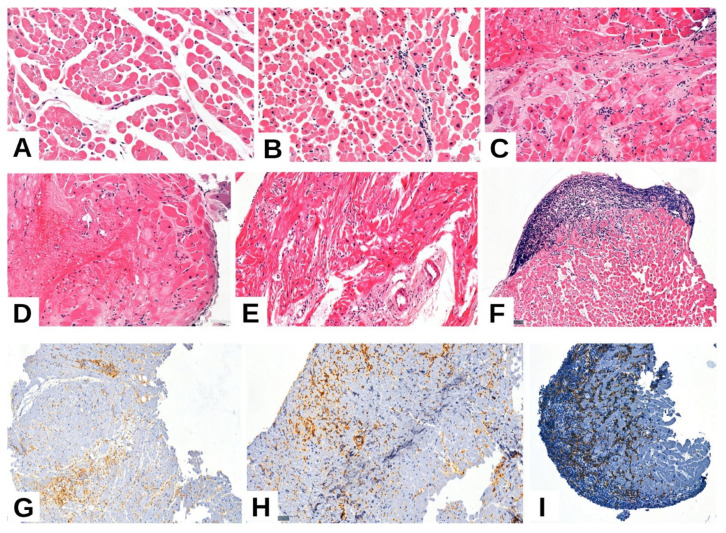
Similar non-rejection related injuries (ACR grade 0): rare interstitial monocytes, isolated phenomena of premiocytolysis and extensive areas of quasi-normal-appearing myocardium (**A**). ACR grade 1R: coagulation necrosis, myocyte degeneration, minimal interstitial lymphoplasmacytic inflammatory infiltrate (one focus) (**B**). ACR grade 2R: Subendocardial coagulation necrosis and isolated degenerative phenomena: premiocytolysis, vacuolar degeneration, fine granular cytoplasm with moderate inflammatory infiltrate (more than two foci) (**C**). ACR grade 3R: marked myocytic degenerative lesions, diffuse inflammatory infiltrate, interstitial hemorrhage, subendocardial myocyte necrosis (**D**). Vascular changes (mild vasculitis)—perivascular inflammatory infiltrate and thickening of the endothelium (**E**). Quilty effect (**F**). Positive immunohistochemical staining for CD4, CD8, and CD68 antibodies highlights the presence of T lymphocytes and macrophages within the myocardial tissue (**G**–**I**), objective (ob.) 10×, 20×.

Regarding myocardial injury, 23.7% of the cases exhibited only degenerative changes in cardiomyocytes, 27.1% exhibited changes consistent with premyocytolysis, and 49.2% exhibited foci of myocardial necrosis and coagulative necrosis. Regarding inflammation, most cases (71.2%) exhibited isolated inflammatory foci (mild inflammation, up to one focus), while similar numbers exhibited moderate (two foci, 10.2%) and severe (diffuse, 8.5%) inflammation. Vasculitis, which is considered a marker of ACR, was present in only 27.1% of the cases. Regarding AMR, like those with ACR, most cases (83.1%) exhibited no histological or immunopathological evidence of rejection (pAMR, ISHLT grade 0). Of the remaining cases, over half (10.2%) exhibited mild immunopathological and histological changes consistent with pAMR (ISHLT grade 1). Severe pathological features—such as interstitial hemorrhage, capillary fragmentation, mixed inflammatory infiltrates, pyknosis, karyorrhexis, and edema—were observed in only one case. The Quilty effect, defined as the presence of a nodular inflammatory infiltrate, was identified in only a minority of cases, with subendocardial localization observed in 6.8% and endocardial localization observed in 5.1% (Table 1, Figure 1).

### 2.2. MMP2 IHC Expression and Pathological Parameters in Cardiac Allograft Rejection

In the study cohort, MMP2 expression exhibited significant associations with multiple pathological, morphological, and IHC parameters. A higher degree of pAMR was associated with increased nuclear and cytoplasmic MMP2 expression (*p* = 0.0002). Notably, a significant association was observed between >50% cytoplasmic MMP2 expression and mild to moderate forms of AMR (OR = 7.47, 95% CI = 1.71–32.68). Negativity for MMP2 expression was observed predominantly in cases without AMR.

A similar positive association was observed in cases with ACR (*p* = 0.0050). A substantial proportion of cases with mild to moderate ACR exhibited positivity for cytoplasmic MMP2 expression, particularly for <50% expression (OR = 7.47, 95% CI = 1.71–32.68), indicating a significant association. Additionally, <50% nuclear positivity was observed in cases with severe ACR (OR = 4.71, 95% CI = 0.18–126.91); however, the wide 95% CI suggests a potential lack of robust statistical significance. Also, negativity for MMP2 was observed predominantly in cases without ACR.

Regarding the endothelial IHC markers, both nuclear and cytoplasmic expression of MMP2 were significantly associated with CD31 (*p* = 0.0100), CD34 molecule (CD34, *p* = 0.0060), and epidermal growth factor receptor (EGFR, *p* = 0.0100) expression. Regarding immunoglobulins, only immunoglobulin M (IgM)—not immunoglobulin A (IgA)—was significantly associated with MMP2 expression (*p* = 0.0100).

**Figure 2:**
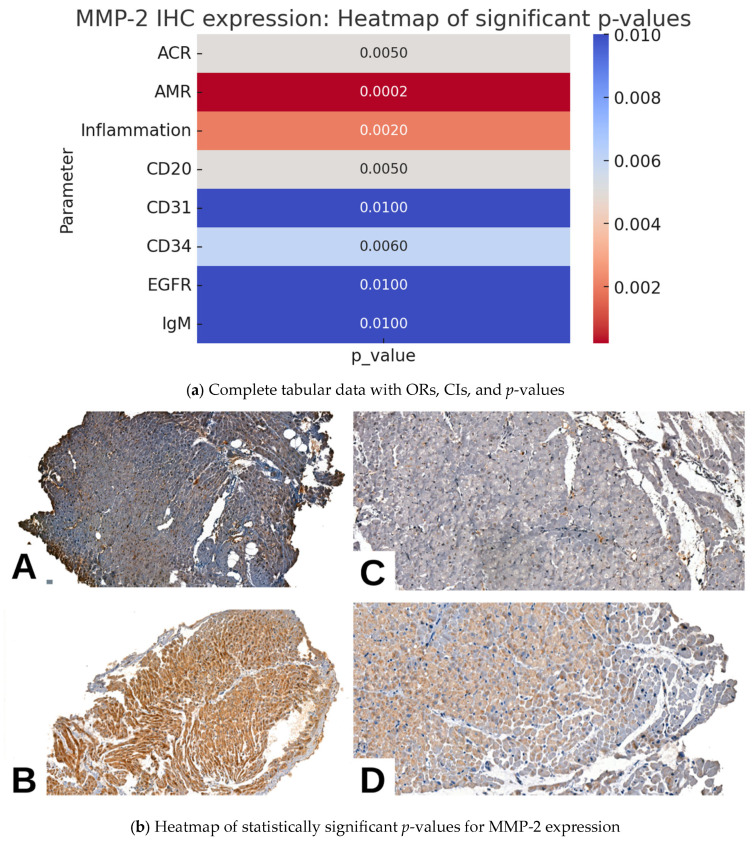
Immunohistochemical expression of MMP-2 in myocardial tissue. Nuclear positivity in more than 50% of cardiomyocytes (**A**). Cytoplasmic positivity in more than 50% (**B**). Nuclear positivity in less than 50% (**C**). Cytoplasmic positivity in less than 50% (**D**) ob. 10× 20×.

Although MMP2 expression was significantly associated with the degree of pAMR, when examined alone, associations with specific immune cell markers linked to humoral rejection—CD4, cluster of differentiation 8 (CD8), and CD68—did not reach statistical significance (*p* = 0.0700, *p* = 0.0700, and *p* = 0.1900, respectively). The only individual IHC surrogate marker for humoral rejection that exhibited a significant association with MMP2 expression was CD20 (*p* = 0.0050). Moreover, the Quilty effect and vasculitis did not exhibit significant associations with MMP2 expression (Table 2, Figure 2).

### 2.3. MMP9 IHC Expression and Pathological Parameters in Cardiac Allograft Rejection

In the study cohort, MMP9 expression was predominantly nuclear, and significant associations were observed with several pathological and IHC parameters. A higher degree of ACR was significantly associated with increased MMP9 nuclear expression (*p* = 0.0007). Notably, mild ACR was more frequently observed in cases with <50% nuclear positivity, while moderate ACR was predominantly associated with >50% nuclear positivity. However, despite elevated ORs (<50%: OR = 2.95, >50%, OR = 1.42), the wide 95% CIs (0.85–10.22 and 0.32–6.27, respectively) limit the statistical significance of these findings.

Regarding AMR, >50% MMP9 nuclear expression exhibited a significant association with mild AMR (OR = 9.07, 95% CI = 1.01–81.16, *p* = 0.05), suggesting a potential diagnostic role. In addition, <50% nuclear positivity exhibited a positive trend (OR = 2.27), although it did not reach statistical significance.

Unlike MMP2, MMP9 exhibited individual significant associations with CD4 (*p* = 0.0300), CD8 (*p* = 0.0300), and CD20 (*p* = 0.0010) expression but not with CD68 expression (*p* = 0.1900). Additionally, vascular markers CD31, CD34, and EGFR exhibited significant associations with MMP9 expression (*p* = 0.0300), similar to those observed with MMP2 expression. Moreover, both the studied immunoglobulins (IgA and IgM) exhibited significant associations with MMP9 expression, whereas only IgM exhibited a significant association with MMP2 expression (both *p* = 0.0300).

**Figure 3:**
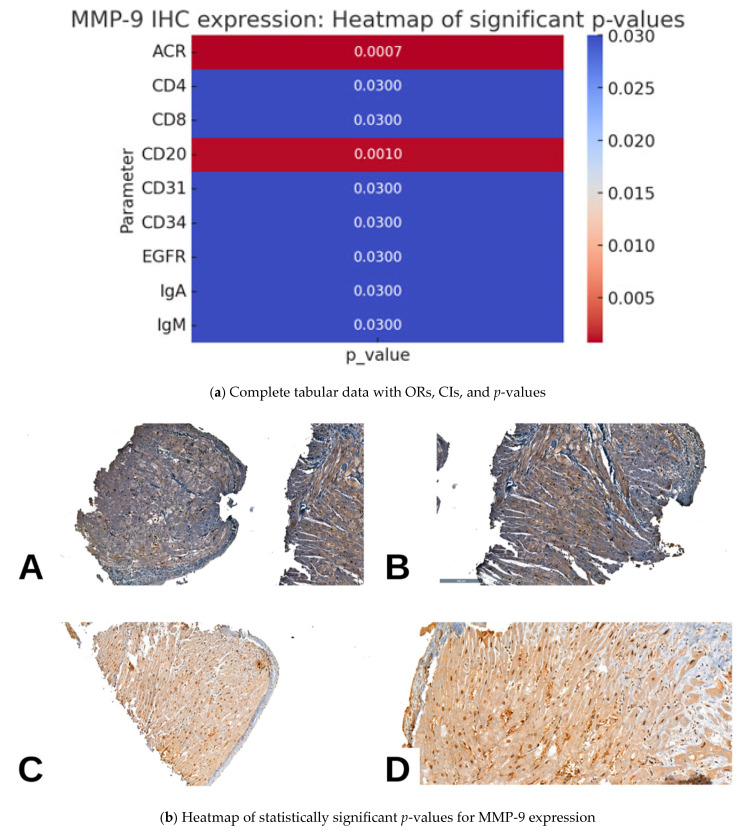
Immunohistochemical expression of MMP-9 in myocardial tissue. Nuclear positivity in less than 50% of cardiomyocytes (**A**,**B**). Nuclear positivity in more than 50% of cardiomyocytes (**C**,**D**) ob. 10× 20×.

Although both MMP2 and MMP9 exhibited multiple significant associations with various morphological and IHC parameters, a trend toward stronger significant associations was observed with surrogate markers of ACR for MMP9 (*p* = 0.0007) and AMR for MMP2 (*p* = 0.0002) (Table 3, Figure 3).

### 2.4. IL-1β IHC Expression and Pathological Parameters in Cardiac Allograft Rejection

In the study cohort, IL-1β expression was significantly associated with multiple pathological and IHC parameters. IL-1β expression was detected in nuclear, cytoplasmic, and membranous compartments, with variable intensity. This marker serves as an indicator for the presence of ACR (*p* = 0.0001) but not AMR (*p* = 0.3710). In addition, <50% nuclear expression exhibited the strongest association with mild to moderate ACR (OR = 8.0, 95% CI = 1.69–37.95), while cytoplasmic and membranous expression exhibited lower, less statistically robust ORs. Moreover, negativity for IL-1β expression was more frequently observed in cases without ACR. In contrast, IL-1β expression was not significantly associated with AMR (*p* = 0.3710), although a moderate trend was noted for <50% nuclear positivity (OR = 6.5, 95% CI = 1.39–30.37), which warrants further investigation in larger cohorts.

The vascular markers (CD31, CD34, and EGFR) exhibited a similar pattern to MMP2 and MMP9, demonstrating positive associations with IL-1β expression (*p* = 0.0022, *p* = 0.0020, and *p* = 0.0030, respectively). The studied immunoglobulins (IgM and IgA) exhibited similar associations with IL-1β expression as they did with MMP9. Specifically, IL-1β and MMP9 expression were both significantly associated with IgA and IgM, whereas MMP2 expression was only significantly associated with IgM.

**Figure 4:**
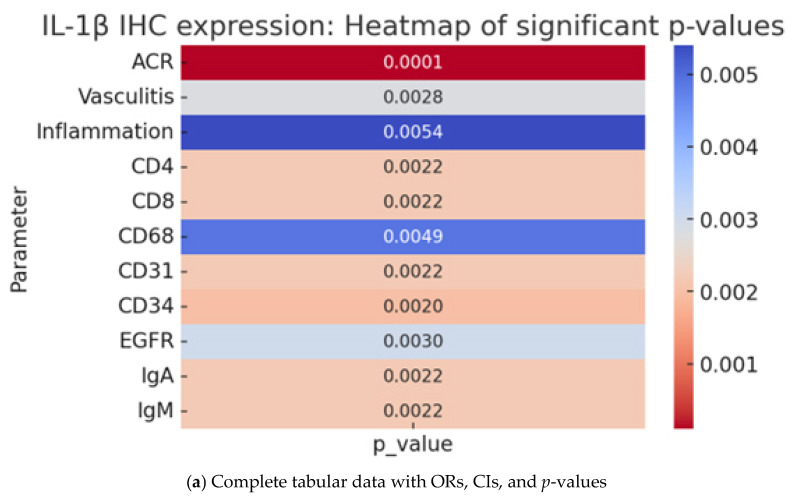

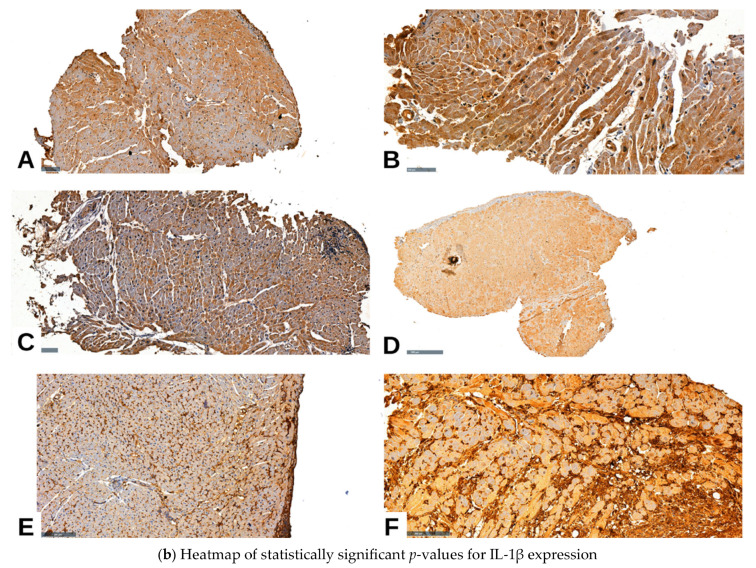
Immunohistochemical expression of IL-1β in myocardial tissue. Nuclear positivity in cardiomyocytes: less than 50% (**A**), more than 50% (**B**). Cytoplasmic positivity: less than 50% (**C**), more than 50% (**D**). Membrane positivity: less than 50% (**E**), more than 50% (**F**), ob. 10×, 20×.

IL-1β expression was significantly associated with vasculitis-related changes (*p* = 0.0028), with >50% nuclear positivity exhibiting a high odds ratio (OR = 25.0, 95% CI = 2.60–240.34), indicating potential involvement in vascular inflammatory damage (Table 4, Figure 4). However, MMP2 and MMP9 expression were not indicative of vasculitis risk (Table 2 and Table 3).

## 3. Discussion

Understanding the complex interplay between immune-mediated injury and tissue remodeling remains a central challenge in cardiac transplantation pathology. While histological grading systems such as those proposed by the ISHLT provide a standardized framework for evaluating rejection, they do not always capture early or evolving immune activity at the molecular level. In this context, IHC markers such as MMPs (e.g., MMP2 and MMP9) and proinflammatory cytokines (e.g., IL-1β) offer a promising adjunctive layer of diagnostic insight. These markers are involved in ECM degradation, leukocyte recruitment, endothelial activation, and immune cell signaling—key processes implicated in both acute and chronic allograft dysfunction.

Our study aimed to evaluate the expression patterns of MMP2, MMP9, and IL-1β in human endomyocardial biopsies and to correlate them with established histopathological parameters, such as ACR, AMR, inflammation, vasculitis, the Quilty effect, and immune marker expression. By integrating clinical, morphological, and IHC data, our study provides one of the first comprehensive assessments of these biomarkers in a human cardiac transplant cohort, addressing a notable gap in the literature. The following discussion contextualizes our findings within current scientific knowledge, highlighting the diagnostic potential and limitations of these markers in transplant pathology.

The clinical and pathological characteristics of our study cohort offer important contextual insights into the interpretation of IHC findings. Most of the included cases were male and over 40 years of age, aligning with the known demographic profiles of heart transplant recipients in Western and European registries [1,3]. Notably, most cases did not present histological evidence of rejection at the time of biopsy, which may reflect the effectiveness of immunosuppression protocols or early surveillance strategies. Among those exhibiting rejection, most showed mild ACR (ISHLT grade 1R), consistent with data indicating that low-grade rejection episodes are the most frequently encountered form in protocol biopsies, particularly within the first year post-transplant [6,26].

Only one case in our study cohort exhibited severe histopathological injury, reinforcing the relatively low incidence of high-grade rejection in well-monitored populations. Regarding myocardial involvement, biopsies with evident cardiac injury most often exhibited coagulative necrosis and myocyte dropout, classical features of active rejection associated with cytotoxic T cell infiltration and local ischemic stress [8,26].

Mild interstitial inflammation was the most frequent inflammatory pattern observed, consistent with previous studies that have identified low-grade inflammation as a common but sometimes nonspecific feature in transplant biopsies [12,26]. Approximately one-third of the biopsies showed vascular involvement in the form of vasculitis, which has been variably associated with early allograft dysfunction and more aggressive rejection phenotypes in other studies [6,22].

Interestingly, the Quilty effect was present in only about 10% of our cases, and predominantly in its subendocardial form. While some studies have proposed that Quilty lesions may represent localized immune activation and may be linked to prior or evolving rejection [25,26], our data do not support a strong association in this cohort. The relatively low incidence observed may reflect the predominantly non-rejection phenotype of the studied population or differences in sampling depth, given that Quilty lesions are more likely to be captured in deeper tissue sections.

Altogether, these histopathological features support the interpretation that our study cohort was dominated by low-grade or absent rejection processes, making it particularly well-suited for identifying subtle IHC variations that precede overt rejection or reflect early immune activation. The integration of these findings with marker expression (e.g., MMP9 and IL-1β) adds a functional dimension to morphologic evaluation and may enhance diagnostic precision in future protocols.

To date, published studies addressing the IHC expression of MMPs (e.g., MMP2 and MMP9) and IL-1β in the context of cardiac allograft rejection remain extremely limited, especially in human clinical cohorts. Most of the existing evidence originates from experimental animal models—predominantly mouse studies—where the upregulation of these markers has been observed during episodes of acute immune-mediated injury or ischemia–reperfusion events [19,21,22,23]. Seminal studies by Simeoni et al. and Palmer et al. investigated the IL-1β and MMP signaling pathways in rodent transplant settings, demonstrating their involvement in graft inflammation and remodeling [19,25]. A recent systematic review reaffirmed this trend, identifying only a few studies that utilized IHC to examine MMPs in transplant rejection, all in animal models [16]. To the best of our knowledge, no large-scale human IHC studies have been published that directly correlate the expression of MMP2, MMP9, and IL-1β with histopathological rejection grading, inflammation, vasculitis, the Quilty effect, or the expression of immune cell markers. Therefore, our study represents one of the first comprehensive evaluations of these markers in human endomyocardial biopsy specimens, aiming to bridge a crucial gap in transplant immunopathology and provide clinically translatable data.

MMP9 is a zinc-dependent endopeptidase implicated in the degradation of ECM proteins and the modulation of immune cell migration. In cardiac allografts, MMP9 plays a pivotal role during acute inflammatory responses, particularly by facilitating leukocyte transmigration across the endothelial barrier and promoting tissue remodeling. Its expression has been linked to immune cell infiltration, vascular injury, and allograft dysfunction, positioning it as a potential biomarker and therapeutic target in transplant rejection [24,27,28]. Our findings show that MMP9 expression is significantly upregulated during both ACR and AMR, especially in biopsies with moderate histological changes. However, the statistical significance was markedly stronger for ACR, suggesting that MMP9 may be more tightly involved in T cell–mediated injury mechanisms. Its association with AMR, although present, did not reach the same level of robustness and appeared closer to the statistical threshold, potentially reflecting a secondary or less dominant role for MMP9 in humoral-mediated injury. This observation supports the notion that MMP9 acts as an effector molecule during active rejection, primarily through its contribution to ECM degradation, disruption of the basement membrane, and facilitation of leukocyte transmigration into the graft tissue.

Previous studies have shown that MMP9 is produced by macrophages, neutrophils, and even endothelial cells in response to proinflammatory stimuli, including IL-1β and tumor necrosis factor (TNF/TNF-α) [26]. Our data align with these findings, confirming that elevated nuclear MMP9 expression is strongly associated with positivity for CD4, CD8, CD68, and endothelial markers such as CD31, CD34, and EGFR, suggesting an intense immune–vascular interaction during rejection episodes. These associations further reinforce the role of MMP9 in immune cell recruitment, endothelial activation, and microvascular injury, particularly within the framework of cell-mediated rejection.

Notably, although less frequent than its nuclear expression, the cytoplasmic expression of MMP9 was associated with more pronounced inflammatory phenotypes. This localization may reflect ongoing intracellular processing, latent enzyme storage, or localized activation of MMP9 in response to tissue stress. While prior studies have documented increased circulating MMP9 levels in patients experiencing acute rejection and associated those elevations with impending allograft dysfunction [28], our tissue-level approach offers higher anatomic resolution and highlights the diagnostic utility of IHC localization in mapping inflammatory burden within defined histopathological compartments.

MMP2, formerly named gelatinase A, is a constitutively expressed zinc-dependent enzyme responsible for degrading key basement membrane components, including type IV collagen and laminin. In the context of cardiac transplantation, MMP2 has been implicated in ECM remodeling, vascular smooth muscle cell activation, and fibrotic progression, particularly in chronic phases of graft remodeling. Unlike MMP9, which is strongly inducible under inflammatory conditions, MMP2 activity is generally more stable and modulated by non-inflammatory cues such as hypoxia, shear stress, and mechanical strain. In animal models, MMP2 has been associated with the pathogenesis of cardiac allograft vasculopathy and interstitial fibrosis, both of which contribute significantly to chronic graft dysfunction and long-term failure [15,24,26].

In our study, MMP2 expression was considerably weaker and showed a more inconsistent pattern compared to MMP9 expression. Cytoplasmic positivity for MMP2 was occasionally observed in mild cases of ACR and AMR, but without significant associations with vasculitis, the Quilty effect, or inflammatory marker expression (CD4, CD8, and CD68). However, a closer analysis of our cohort revealed a notable trend: MMP2 expression was more consistently associated with AMR grades than with ACR, particularly when its cytoplasmic positivity was >50%. This finding suggests a potential role for MMP2 in endothelial activation and humoral immune injury—hallmarks of AMR—possibly through its involvement in capillary basement membrane degradation and vascular remodeling. Although these associations did not always reach robust statistical thresholds, the trend underscores a preferential link between MMP2 activity and AMR pathways, rather than cell-mediated immune responses.

These observations support previous experimental findings suggesting that MMP2 plays a limited role in acute T cell–driven injury and may be more prominently involved in chronic and vascular changes characteristic of humoral rejection [14,15,24]. In addition, while MMP2 and MMP9 share overlapping substrate specificity and both participate in ECM turnover, their regulation and immunopathologic impact differ considerably. MMP2 is less inducible by proinflammatory cytokines such as IL-1β or TNF-α and may reflect basal or reparative activity rather than acute immune activation [25].

Importantly, animal transplant models have demonstrated that knockout of MMP2 leads to attenuated fibrosis and improved graft survival, whereas knockout of MMP9 paradoxically accelerates rejection. This difference highlights the divergent roles of these two MMPs in transplant immunobiology, with MMP2 potentially facilitating late-stage remodeling rather than early immune effector responses [26,28].

Altogether, our findings add to the growing body of evidence that MMP2, although biologically active in the graft microenvironment, is more aligned with humoral pathways and chronic remodeling, rather than serving as a robust marker of ACR. Therefore, its potential value may lie more in prognostic ability, particularly for AMR-associated vasculopathy, than in early diagnostic assessment of rejection episodes. Although our study focused on tissue expression in EMB samples, previous studies have demonstrated that MMPs and IL-1β can also be detected in peripheral blood, where they reflect systemic inflammatory and vascular injury pathways [29,30,31]. This raises the possibility that these molecules could serve as non-invasive serum biomarkers for earlier detection of rejection, potentially complementing donor-specific antibody (DSA) testing before the development of clinical or biopsy-proven rejection. Future studies integrating tissue and circulating levels will be required to confirm this hypothesis.

IL-1β is a central mediator of the innate immune response and a key cytokine in the orchestration of acute inflammation. In the context of cardiac transplantation, IL-1β plays a pivotal role in initiating immune activation within the graft, acting upstream of cellular infiltration, endothelial activation, and subsequent tissue injury. Its involvement has been well documented in both ischemia–reperfusion injury and early rejection phases, where it promotes the recruitment of leukocytes, the activation of endothelial and stromal cells, and the induction of downstream effectors such as MMPs (notably MMP9) [14,20,25]. Despite its recognized role in various inflammatory cardiomyopathies, the specific expression pattern and significance of IL-1β in human cardiac allografts have not been systematically characterized until recently.

Among the three studied markers, IL-1β demonstrated the broadest and most consistent associations with pathological parameters. Its nuclear expression was strongly associated with ACR grades, vasculitis, diffuse inflammation, and positivity for immune markers. IL-1β is a key proinflammatory cytokine known to stimulate endothelial activation, leukocyte recruitment, and fibroblast proliferation [14,20,32]. Our data confirm its early upregulation even in mild ACR (ISHLT grade 1R), suggesting that IL-1β may precede overt histologic rejection. This finding is consistent with previous models, in which IL-1β signaling triggered early graft inflammation and contributed to the downstream activation of MMPs [18,25]. Notably, IL-1β expression was significantly correlated with MMP9 expression but not MMP2 expression, reinforcing the possibility of a sequential IL-1β–MMP-9 axis in acute allograft injury [2,24].

While historically interpreted as a benign or reparative phenomenon, the Quilty effect has recently gained attention for its possible association with immune activation and allograft dysfunction. In our study cohort, IL-1β and MMP9 were more frequently expressed in biopsies showing Quilty lesions, particularly those with subendocardial extension. Although the statistical significance was moderate, this trend supports the hypothesis that the Quilty effect may be an indicator of localized immune activation, particularly of the T- and B-cell subsets, and not only a histologic curiosity [17,27].

Interestingly, vasculitis—a morphologic indicator of acute immune damage—was significantly associated only with IL-1β expression. While MMP9 expression showed an upward trend in these cases, it did not reach statistical significance, possibly due to sample size limitations. Again, MMP2 showed no consistent involvement. These findings underscore the role of IL-1β in endothelial and perivascular injury, reinforcing its central place in early rejection mechanisms [20,21]. Our results are consistent with evidence from other immune-mediated vascular pathologies. For example, a recent study in antiphospholipid syndrome demonstrated that heparanase activity promotes tissue factor overexpression in endothelial cells and platelets through IL-1 receptor-associated kinase (IRAK1) and NF-κB signaling, thereby linking IL-1β with extracellular matrix–remodeling enzymes and vascular immune activation [33]. This supports the broader relevance of IL-1β and MMPs as central mediators of immune–vascular injury, not only in cardiac allograft rejection but also in other inflammatory vascular disorders.

From a translational perspective, our results suggest that IHC staining for IL-1β and MMP9 could serve as valuable adjunct markers in evaluating ambiguous or borderline biopsy findings. In settings where classical histology (e.g., absence of myocyte necrosis or capillary injury) fails to capture immune activation, these markers may help stratify patients who might benefit from therapeutic escalation. Furthermore, serial biopsies in such patients could be used to monitor dynamic changes in marker expression and guide personalized immunosuppression.

From a therapeutic perspective, MMP9 and IL-1β are both being considered as potential drug targets. Preclinical data have shown that MMP9 inhibitors can delay rejection and reduce fibrosis in mouse models [22,23]. In addition, IL-1β blockade (e.g., with anakinra) has been trialed in post-myocardial infarction remodeling and could be explored in transplant settings [18,20]. Given their strong expression in our study and their early involvement in rejection pathogenesis, these markers may also serve as candidate biomarkers in future clinical trials aimed at improving graft survival.

### Future Directions

Given the limited number of human studies investigating IHC markers in cardiac allograft rejection, future research should aim to expand upon our findings through larger, multicenter cohorts and prospective designs. A more detailed characterization of the temporal dynamics of MMP9, MMP2, and IL-1β expression during the post-transplant period may yield important diagnostic and prognostic insights. Additionally, integrating IHC data with molecular tools, such as transcriptomics or spatial proteomics, could enhance the sensitivity of early rejection detection and clarify the mechanistic roles of these biomarkers in different rejection subtypes. Standardization of IHC scoring criteria and correlation with long-term graft outcomes may also support clinical implementation. Finally, the potential value of these markers as therapeutic targets, particularly for early intervention in subclinical rejection or chronic allograft vasculopathy, warrants continued investigation. Another important direction for future research will be to investigate the relationship between MMP2, MMP9, and IL-1β expression and the development of coronary allograft vasculopathy (CAV). Given the vascular associations observed in our study, these molecules may play a role not only in acute immune-mediated injury but also in chronic vascular remodeling after transplantation. Prospective studies integrating tissue expression, serum levels, and longitudinal follow-up with imaging-based assessment of CAV could provide valuable insights into their potential as predictive biomarkers of chronic graft vasculopathy.

## 4. Materials and Methods

### 4.1. Study Participants

Our study enrolled 59 patients with cardiac transplants performed at the Emergency Institute for Cardiovascular Diseases and Tranplant in Târgu Mureș, Romania, who were diagnosed with cellular and humoral rejection between 2020 and 2023 at the Department of Pathology of the Clinical County Emergency Hospital in Târgu Mureș, Romania. The inclusion criteria were as follows: cardiac transplant with different grades of cellular rejection (ISHLT grades 0–3R), with or without associated humoral rejection, diagnosed on biopsy specimens. The exclusion criteria were as follows: tissues unsuitable for processing and death shortly after surgery, before it was possible to collect endomyocardial tissue biopsies. At our center, endomyocardial biopsies are routinely performed at one month, six months, and one year post-transplantation, followed by annual biopsies during the first five years post-transplant. The biopsy specimens analyzed in this study were obtained in accordance with this institutional surveillance protocol. The processing of the cases was approved by the Ethical Committee of the Clinical County Emergency Hospital in Târgu Mureș, Romania (approval no 15373/16.06.2021).

### 4.2. Immunohistochemical Analysis

For all cases, the available slides with different grades of cellular rejection were reanalyzed. We aimed to establish the staging according to the most recent edition of cardiac rejection proposed by the ISHLT (Table 5).

Immunopathologic findings characteristic of acute AMR include positive immunofluorescent staining for the split products of complement C4A (C4d) and complement C3 (C3d), as well as the DR isotype of HLA (HLA-DR), or positive immunoperoxidase staining for C4d and CD68 (or C3d) [34].

The Quilty effect, frequently associated with ACR, was investigated based on the updated definition, which describes it as a circumscribed inflammatory infiltrate adjacent to the endocardium. This infiltrate consists of a core of B cells and a variable number of plasma cells, macrophages, and dendritic cells, along with small capillaries surrounded by T cells. It may also extend into the myocardium and can be transmural, involving the epicardial tissue [27].

We performed immunohistochemical (IHC) analysis for MMP2, MMP9, and IL-1β in all 59 cases using 4-μm-thick sections prepared from formalin-fixed, paraffin-embedded tissue with an automated immunostainer (Benchmark GX; Ventana Medical Systems, Inc., Tucson, AZ, USA). All reagents and incubation times were selected according to the directions given in the antibody package inserts (Table 6). The slides were developed using the OmniMap DAB (3,3′-Diaminobenzidine) Detection Kit (Ventana Medical Systems, Inc.) and were counterstained with Hematoxylin. Human breast carcinoma was used as an external positive control for MMP2 and MMP9, and normal lung tissue was used as an external positive control for IL-1β. All slides were independently evaluated by two experienced pathologists, blinded to clinical and outcome data. Immunohistochemical positivity was defined using a cutoff of >50% stained cardiomyocytes cells for each marker. In cases of discordance, results were re-examined jointly, and consensus was reached. This approach minimized inter-observer variability and enhanced reproducibility. Staining patterns were defined as nuclear or cytoplasmic for MMP2 and MMP9, and as membranous, cytoplasmic, or nuclear for IL-1β (Table 2, Table 3 and Table 4).

### 4.3. Statistical Analysis

For improved clarity, each statistical dataset is presented as a composite table with two subpanels. specifically, in Table 2, Table 3 and Table 4, panel (a) contains the complete statistical dataset (distribution, OR, CI, *p*-values), while panel (b) provides a heatmap visualization of the significant *p*-values for each marker.

## 5. Conclusions

Our study provides one of the first systematic IHC assessments of MMP2, MMP9, and IL-1β expression in human cardiac transplant biopsies, highlighting their differential associations with histopathological features of rejection and inflammation. Among these three markers, IL-1β exhibited the strongest and most consistent associations with acute rejection, inflammation, and vascular injury. MMP9 was also significantly associated with immune activation, particularly in ACR, while MMP2 demonstrated a weaker and more variable expression pattern, with preferential alignment to humoral rejection. These findings underscore the potential utility of selected tissue biomarkers in complementing conventional histologic criteria, offering a more nuanced understanding of the immune and vascular responses in the transplanted myocardium. Although preliminary, these results encourage the integration of functional immunomarkers into routine diagnostic workflows and support the need for further studies to validate their clinical utility.

## Figures and Tables

**Table 1:** Clinicopathological features of the evaluated cases.

	Parameter	n = 59
Mean Age (Years)	56 (±15.8) Years	
Gender	Males	48 (81.4%)
	Females	11 (18.6%)
Age (years)	<40	29 (49.2)
	≥40	30 (50.8%)
Quilty effect	Absent	52 (88.1%)
	Subendocardial	4 (6.8%)
	Endocardial	3 (5.1%)
Fibrosis	Absent	13 (22.0%)
	Mild	25 (42.4%)
	Moderate	7 (11.9%)
	Severe	15 (25.4%)
Vasculitis	Present	16 (27.1%)
	Absent	43 (72.9%)
Inflammation	Absent	5 (8.5%)
	Mild (1 focus)	42 (71.2%)
	Moderate (2 foci)	6 (10.2%)
	Severe (diffuse)	5 (8.5%)
Cardiomyocyte damage	Degenerative lesions	14 (23.7%)
	Premiocitolisis	16 (27.1%)
	Myocite necrosis, coagulative necrosis	29 (49.2%)
Antibody mediated rejection (AMR)	Histologic and immunopathologic studies are both absent (pAMR0)	49 (83.1%)
	Histologic findings or immunopathologic findings are present (pAMR1)	6 (10.2%)
	Histologic and immunopathologic findings are both present (pAMR2)	3 (5.1%)
	Severe pathologic AMR (pAMR3) (interstitial hemorrhage, capillary fragmentation, mixed inflammatory infiltrates, pyknosis, karyorrhexis, edema)	1 (1.7%)
Acute cellular rejection (ACR)	Absent (0)	48 (81.6%)
	Mild (1R)	8 (13.6%)
	Moderate (2R)	2 (3.4%)
	Severe (3R)	1 (1.7%)

**Table 2:** Distribution of MMP-2 immunohistochemical expression according to pathological and immunohistochemical parameters.

Parameter		MMP-2 IHC Expression (n = 59)					*p* Value
		Negative	Nuclear Positivity under 50%	Nuclear Positivity over 50%	Citoplasm Positivity under 50%	Citoplasm Positivity over 50%	
**Acute Cellular Rejection (ACR)**	Absent	16 (27.1%)	0	4 (6.8%)	3 (5.1%)	1 (1.7%)	**0.005**
	Mild	10 (16.9%)	0	1 (1.7%)	13 (22.0%)	9 (15.3%)	
	Moderate	0	0	0	0	0	
	Severe	0	1 (1.7%)	0	1 (1.7%)	0	
**OR vs. Negative** **CI**		Ref	4.71	0.40	7.47	14.4	
			0.18–126.91	0.04–4.11	1.71–32.68	1.58–135.52	
**Antibody-mediated Rejection (AMR)**	Absent	17 (28.8%)	0	4 (6.8%)	3 (5.1%)	1 (1.7%)	**0.0002**
	Mild	10 (16.9%)	0	1 (1.7%)	13 (22.0%)	9 (15.3%)	
	Moderate	0	1 (1.7%)	0	0	0	
	Severe	0	0	0	1 (1.7%)	0	
**OR vs. Negative** **CI**		Ref	4.71	0.4	7.47	14.4	
			0.18–126.91	0.04–4.11	1.71–32.68	1.58–131.52	
**Vasculitis**	Absent	14 (23.7%)	0	3 (5.1%)	5 (8.5%)	1 (1.7%)	0.06
	Present	12 (20.3%)	1 (1.7%)	2 (3.4%)	12 (20.3%)	10 (16.9%)	
**OR vs. Negative** **CI**		Ref	3.48	0.78	2.82	11.67	
			0.13–93.31	0.11–5.46	0.77–10.25	1.30–104.82	
**Inflammation**	Absent	032w	0	1 (1.7%)	0	0	**0.002**
	Low	21 (35.6%)	0	3 (5.1%)	7 (11.9%)	6 (10.2%)	
	Moderate	1 (1.7%)	0	0	3 (5.1%)	4 (6.8%)	
	Severe	4 (6.8%)	1 (1.7%)	1 (1.7%)	7 (11.9%)	0	
**OR vs. Negative** **CI**		Ref	0.23	0.08	2.08	1.62	
			0.0–17.06	0.0–2.99	0.04–116.87	0.03–91.82	
**Quilty Effect**	Absent	25 (42.4%)	1 (1.7%)	4 (6.8%)	11 (18.6%)	4 (6.8%)	0.2299
	Subendocardial	0	0	1 (1.7%)	3 (5.1%)	6 (10.2%)	
	Endocardial	1 (1.7%)	0	0	3 (5.1%)	0	
**OR vs. Negative** **CI**		Ref	5.67	6.25	13.64	37.5	
			0.15–207.27	0.32–121.34	1.46–127.15	3.52–399.38	
**CD4**	Negative	12 (20.3%)	0	2 (3.4%)	2 (3.4%)	1 (1.7%)	0.07
	Positive	14 (23.7%)	0	2 (3.4%)	2 (3.4%)	1 (1.7%)	
**OR vs. Negative** **CI**		Ref	1.07	1.08	1.08	1.08	
			0.02–58.03	0.13–8.8	0.13–8.8	0.06–19.05	
**CD8**	Negative	12 (20.3%)	0	2 (3.4%)	2 (3.4%)	1 (1.7%)	0.07
	Positive	14 (23.7%)	1 (1.7%)	3 (5.1%)	15 (25.4%)	9 (15.3%)	
**OR vs. Negative** **CI**		Ref	6.33	3.23	16.15	19.38	
			0.24–165.89	0.48–21.74	3.21–81.25	2.22–169.47	
**CD68**	Negative	13 (22.0%)	0	2 (3.4%)	5 (8.5%)	1 (1.7%)	0.19
	Positive	13 (22.0%)	1 (1.7%)	3 (5.1%)	12 (20.3%)	9 (15.3%)	
**OR vs. Negative** **CI**		Ref	4.12	2.07	3.31	12.43	
			0.16–106.01	0.32–13.51	1.01–10.84	1.46–105.74	
**CD20**	Negative	25 (42.4%)	1 (1.7%)	4 (6.8%)	11 (18.6%)	4 (6.8%)	**0.005**
	Positive	1 (1.7%)	0	1 (1.7%)	6 (10.2%)	6 (10.2%)	
**OR vs. Negative** **CI**		Ref	0.12	0.09	0.19	0.51	
			0–3	0.01–0.82	0.06–0.59	0.13–2.06	
**CD31**	Negative	14 (23.7%)	0	2 (3.4%)	2 (3.4%)	1 (1.7%)	**0.01**
	Positive	12 (20.3%)	1 (1.7%)	3 (5.1%)	15 (25.4%)	9 (15.3%)	
**OR vs. Negative** **CI**		Ref	5.78	2.92	14.62	17.55	
			0.23–148.31	0.45–18.95	3.04–70.36	2.07–148.45	
**CD34**	Negative	14 (23.7%)	0	2 (3.4%)	1 (1.7%)	1 (1.7%)	**0.006**
	Positive	12 (20.3%)	1 (1.7%)	3 (5.1%)	16 (27.1%)	9 (15.3%)	
**OR vs. Negative** **CI**		Ref	6.73	3.42	36.44	20.5	
			0.26–173.08	0.52–22.24	4.49–296.11	2.41–174.07	
**EGFR**	Negative	18 (30.5%)	0	2 (3.4%)	6 (10.2%)	1 (1.7%)	**0.01**
	Positive	8 (13.6%)	1 (1.7%)	3 (5.1%)	11 (18.6%)	9 (15.3%)	
**OR vs. Negative** **CI**		Ref	3.55	1.78	2.17	10.67	
			0.14–90.59	0.28–11.43	0.71–6.65	1.27–89.63	
**IgA**	Negative	12 (20.3%)	0	2 (3.4%)	2 (3.4%)	1 (1.7%)	0.07
	Positive	14 (23.7%)	1 (1.7%)	3 (5.1%)	15 (25.4%)	9 (15.3%)	
**OR vs. Negative** **CI**		Ref	6.23	3.16	15.79	6.23	
			0.24–160.04	0.49–20.5	3.27–76.14	2.24–160.56	
**IgM**	Negative	14 (23.7%)	0	2 (3.4%)	2 (3.4%)	1 (1.7%)	**0.01**
	Positive	12 (20.3%)	1 (1.7%)	3 (5.1%)	15 (25.4%)	9 (15.3%)	
**OR vs. Negative** **CI**		Ref	6.23	3.16	15.79	18.95	
			0.24–160.04	0.49–20.5	3.27–76.14	2.24–160.56	

**Table 3:** Distribution of MMP-9 immunohistochemical expression according to pathological and immunohistochemical parameters.

Parameter		MMP-9 IHC Expression (n = 59)			*p* Value
		Negative	Nuclear Positivity under 50%	Nuclear Positivity over 50%	
**Acute Cellular Rejection (ACR)**	Absent	17 (28.8%)	5 (8.5%)	4 (6.8%)	**0.0007**
	Mild	14 (23.7%)	7 (11.9%)	0	
	Moderate	1 (1.7%)	5 (8.5%)	5 (8.5%)	
	Severe	0	1 (1.7%)	0	
**OR vs. Negative** **CI**		Ref	2.95	1.42	
			0.85–10.22	0.32–6.27	
**Antibody-mediated Rejection (AMR)**	Absent	17 (28.8%)	6 (10.2%)	1 (1.7%)	**0.05**
	Mild	15 (25.4%)	11 (18.6%)	7 (11.9%)	
	Moderate	0	0	0	
	Severe	0	1 (1.7%)	1 (1.7%)	
**OR vs. Negative** **CI**		Ref	2.27	9.07	
			0.68–7.53	1.01–81.16	
**Vasculitis**	Absent	15 (25.4%)	6 (10.2%)	2 (3.4%)	0.34
	Present	17 (28.8%)	12 (20.3%)	7 (11.9%)	
**OR vs. Negative** **CI**		Ref	1.76	3.09	
			0.53–5.87	0.55–17.21	
**Inflammation**	Absent	0	1 (1.7%)	0	0.12
	Low	23 (39.0%)	9 (15.3%)	5 (8.5%)	
	Moderate	1 (1.7%)	4 (6.8%)	3 (5.1%)	
	Severe	8 (13.6%)	4 (6.8%)	1 (1.7%)	
**OR vs. Negative** **CI**		Ref	0.18	0.29	
			0.01–4.64	0.01–15.74	
**Quilty Effect**	Absent	30 (50.8%)	11 (18.6%)	4 (6.8%)	0.21
	Subendocardial	1 (1.7%)	4 (6.8%)	5 (8.5%)	
	Endocardial	1 (1.7%)	3 (5.1%)	0	
**OR vs. Negative** **CI**		Ref	9.55	18.75	
			1.72–53.13	2.68–130.95	
**CD4**	Negative	15 (25.4%)	3 (5.1%)	1 (1.7%)	**0.03**
	Positive	17 (28.8%)	15 (25.4%)	8 (13.6%)	
**OR vs. Negative** **CI**		Ref	4.41	7.06	
			1.07–18.27	0.79–63.18	
**CD8**	Negative	15 (25.4%)	3 (5.1%)	1 (1.7%)	**0.03**
	Positive	17 (28.8%)	15 (25.4%)	8 (13.6%)	
**OR vs. Negative** **CI**		Ref	4.41	7.06	
			1.07–18.27	0.79–63.18	
**CD68**	Negative	14 (23.7%)	6 (10.2%)	1 (1.7%)	0.19
	Positive	18 (30.5%)	12 (20.3%)	8 (13.6%)	
**OR vs. Negative** **CI**		Ref	1.56	6.22	
			0.47–5.18	0.69–55.77	
**CD20**	Negative	30 (50.8%)	11 (18.6%)	4 (6.8%)	**0.001**
	Positive	2 (3.4%)	7 (11.9%)	5 (8.5%)	
**OR vs. Negative** **CI**		Ref	9.55	18.78	
			1.72–53.13	2.68–130.95	
**CD31**	Negative	15 (25.4%)	3 (5.1%)	1 (1.7%)	**0.03**
	Positive	17 (28.8%)	15 (25.4%)	8 (13.6%)	
**OR vs. Negative** **CI**		Ref	4.41	7.06	
			1.07–18.27	0.79–63.18	
**CD34**	Negative	15 (25.4%)	3 (5.1%)	1 (1.7%)	**0.03**
	Positive	17 (28.8%)	15 (25.4%)	8 (13.6%)	
**OR vs. Negative** **CI**		Ref	4.41	7.06	
			1.07–18.27	0.79–63.18	
**EGFR**	Negative	19 (32.2%)	8 (13.6%)	1 (1.7%)	**0.03**
	Positive	13 (22.0%)	10 (16.9%)	8 (13.6%)	
**OR vs. Negative** **CI**		Ref	1.83	11.69	
			0.57–5.87	1.3–105.03	
**IgA**	Negative	15 (25.4%)	3 (5.1%)	1 (1.7%)	**0.03**
	Positive	17 (28.8%)	15 (25.4%)	8 (13.6%)	
**OR vs. Negative** **CI**		Ref	4.41	7.06	
			1.07–18.27	0.79–63.18	
**IgM**	Negative	15 (25.4%)	3 (5.1%)	1 (1.7%)	**0.03**
	Positive	17 (28.8%)	15 (25.4%)	8 (13.6%)	
**OR vs. Negative** **CI**		Ref	4.41	7.06	
			1.07–18.27	0.79–63.18	

**Table 4:** Distribution of IL-1β immunohistochemical expression according to pathological and immunohistochemical parameters.

Parameter		IL-1 IHC Interpretation (n = 59)							*p* Value
		Negative	Nuclear Positivity under 50%	Nuclear Positivity over 50%	Citoplasm Positivity under 50%	Citoplasm Positivity over 50%	Membranous Positivity under 50%	Membranous Positivity over 50%	
**Acute Cellular Rejection (ACR)**	Absent	14 (23.7%)	3 (5.1%)	4 (6.8%)	3 (5.1%)	0	2 (3.4%)	0	**0.0001**
	Mild	4 (6.8%)	8 (13.6%)	5 (8.5%)	2 (3.4%)	0	1 (1.7%)	1 (1.7%)	
	Moderate	2 (3.4%)	4 (6.8%)	2 (3.4%)	0	0	2 (3.4%)	1 (1.7%)	
	Severe	1 (1.7%)	0	0	0	0	0	0	
**OR vs. Negative** **CI**		Ref	8.0	3.5	1.33	1.93	3.0	9.67	
			1.69–37.95	0.76–16.12	0.18–9.91	0.03–107.46	0.4–22.3	0.41–228.26	
**Antibody-mediated Rejection (AMR)**	Absent	13 (22.0%)	3 (5.1%)	3 (5.1%)	3 (5.1%)	0	1 (1.7%)	1 (1.7%)	0.371
	Mild	7 (11.9%)	11 (18.6%)	8 (13.6%)	2 (3.4%)	0	4 (6.8%)	1 (1.7%)	
	Moderate	0	0	0	0	0	0	0	
	Severe	1 (1.7%)	1 (1.7%)	0	0	0	0	0	
**OR vs. Negative** **CI**		Ref	6.5	4.33	1.08	1.59	6.5	1.62	
			1.39–30.37	0.88–21.31	0.15–7.96	0.03–87.84	0.61–68.96	0.09–29.78	
**Vasculitis**	Absent	15 (25.4%)	5 (8.5%)	1 (1.7%)	2 (3.4%)	0	0	0	**0.0028**
	Present	6 (10.2%)	10 (16.9%)	10 (16.9%)	3 (5.1%)	0	5 (8.5%)	2 (3.4%)	
**OR vs. Negative** **CI**		Ref	5.0	25.0	3.75	2.5	25.0	10.0	
			1.19–20.92	2.6–240.34	0.5–28.39	0.04–141.02	1.18–531.79	0.39–255.43	
**Inflammation**	Absent	0	0	0	0	0	1 (1.7%)	0	**0.0054**
	Low	15 (25.4%)	9 (15.3%)	8 (13.6%)	4 (6.8%)	0	1 (1.7%)	0	
	Moderate	3 (5.1%)	1 (1.7%)	0	0	0	2 (3.4%)	2 (3.4%)	
	Severe	3 (5.1%)	5 (8.5%)	3 (5.1%)	1 (1.7%)	0	1 (1.7%)	0	
**OR vs. Negative** **CI**		Ref	0.71	0.52	0.24	0.02	0.1	0.1	
			0.01–38.06	0.01–28.24	0–13.52	0–2.95	0–3.35	0–6.22	
**Quilty Effect**	Absent	18 (30.5%)	12 (20.3%)	6 (10.2%)	5 (8.5%)	0	3 (5.1%)	1 (1.7%)	0.3028
	Subendocardial	2 (3.4%)	2 (3.4%)	4 (6.8%)	0	0	2 (3.4%)	0	
	Endocardial	1 (1.7%)	1 (1.7%)	1 (1.7%)	0	0	0	1 (1.7%)	
**OR vs. Negative** **CI**		Ref	1.5	5.0	0.6	6.0	4.0	6.0	
			0.26–8.71	0.91–27.47	0.03–14.05	0.1–364.24	0.46–34.92	0.29–124.1	
**CD4**	Negative	13 (22.0%)	2 (3.4%)	1 (1.7%)	3 (5.1%)	0	0	0	**0.0022**
	Positive	8 (13.6%)	13 (22.0%)	10 (16.9%)	2 (3.4%)	0	5 (8.5%)	2 (3.4%)	
**OR vs. Negative** **CI**		Ref	10.56	16.25	1.08	1.62	16.25	6.5	
			1.87–59.56	1.74–152.09	0.15–7.96	0.03–90.3	0.78–338.89	0.26–162.96	
**CD8**	Negative	13 (22.0%)	2 (3.4%)	1 (1.7%)	3 (5.1%)	0	0	0	**0.0022**
	Positive	8 (13.6%)	13 (22.0%)	10 (16.9%)	2 (3.4%)	0	5 (8.5%)	2 (3.4%)	
**OR vs. Negative** **CI**		Ref	10.56	16.25	1.08	1.62	16.25	6.5	
			1.87–59.56	1.74–152.09	0.15–7.96	0.03–90.3	0.78–338.89	0.26–162.96	
**CD68**	Negative	14 (23.7%)	3 (5.1%)	1 (1.7%)	2 (3.4%)	0	0	1 (1.7%)	0.0049
	Positive	7 (11.9%)	12 (20.3%)	10 (16.9%)	3 (5.1%)	0	5 (8.5%)	1 (1.7%)	
**OR vs. Negative** **CI**		Ref	8.0	20.0	3.0	2.0	20.0	2.0	
			1.69–37.95	2.11–189.18	0.4–22.3	0.04–111.8	0.95–420.36	0.11–36.95	
**CD20**	Negative	18 (30.5%)	12 (20.3%)	6 (10.2%)	5 (8.5%)	0	3 (5.1%)	1 (1.7%)	0.2156
	Positive	3 (5.1%)	3 (5.1%)	5 (8.5%)	0	0	2 (3.4%)	1 (1.7%)	
**OR vs. Negative** **CI**		Ref	1.5	5.0	0.6	6.0	4.0	6.0	
			0.26–8.71	0.91–27.47	0.03–14.05	0.1–364.24	0.46–34.92	0.29–124.1	
**CD31**	Negative	13 (22.0%)	2 (3.4%)	1 (1.7%)	3 (5.1%)	0	0	0	**0.0022**
	Positive	8 (13.6%)	13 (22.0%)	10 (16.9%)	2 (3.4%)	0	5 (8.5%)	2 (3.4%)	
**OR vs. Negative** **CI**		Ref	10.56	16.25	1.08	1.62	16.25	6.5	
			1.87–59.56	1.74–152.09	0.15–7.96	0.03–90.3	0.78–338.89	0.26–162.96	
**CD34**	Negative	13 (22.0%)	2 (3.4%)	1 (1.7%)	3 (5.1%)	0	0	0	**0.002**
	Positive	8 (13.6%)	13 (22.0%)	10 (16.9%)	2 (3.4%)	0	5 (8.5%)	2 (3.4%)	
**OR vs. Negative** **CI**		Ref	10.56	16.25	1.08	1.62	16.25	6.5	
			1.87–59.56	1.74–152.09	0.15–7.96	0.03–90.3	0.78–338.89	0.26–162.96	
**EGFR**	Negative	15 (25.4%)	7 (11.9%)	1 (1.7%)	4 (6.8%)	0	0	1 (1.7%)	**0.003**
	Positive	6 (10.2%)	8 (13.6%)	10 (16.9%)	1 (1.7%)	0	5 (8.5%)	1 (1.7%)	
**OR vs. Negative** **CI**		Ref	2.86	25.0	0.62	2.5	25.0	2.5	
			0.71–11.44	2.6–240.34	0.06–6.8	0.04–141.02	1.18–531.79	0.13–46.77	
**IgA**	Negative	13 (22.0%)	2 (3.4%)	1 (1.7%)	3 (5.1%)	0	0	0	**0.0022**
	Positive	8 (13.6%)	13 (22.0%)	10 (16.9%)	2 (3.4%)	0	5 (8.5%)	2 (3.4%)	
**OR vs. Negative** **CI**		Ref	10.56	16.25	1.08	1.62	16.25	6.5	
			1.87–59.56	1.74–152.09	0.15–7.96	0.03–90.3	0.78–338.89	0.26–162.96	
**IgM**	Negative	13 (22.0%)	2 (3.4%)	1 (1.7%)	3 (5.1%)	0	0	0	**0.0022**
	Positive	8 (13.6%)	13 (22.0%)	10 (16.9%)	2 (3.4%)	0	5 (8.5%)	2 (3.4%)	
**OR vs. Negative** **CI**		Ref	10.56	16.25	1.08	1.62	16.25	6.5	
			1.87–59.56	1.74–152.09	0.15–7.96	0.03–90.3	0.78–338.89	0.26–162.96	

**Table 5:** Grading of Acute Cellular and Antibody-Mediated Rejection According to the Latest ISHLT Classification.

	ACR	AMR
Grade 0	No rejection	Negative histologic and immunopathologic findings
Grade 1	1R, mild rejection: Interstitial and/or perivascular infiltrate with up to 1 focus of myocyte damage	Presence of positive histologic and immunopathologic findings
Grade 2	2 R, moderate rejection: 2 or more foci of infiltrates with associated myocyte damage	Presence of both histologic and immunopathologic findings
Grade 3	3R severe: Diffuse infiltrate with multifocal myocyte damage, with or without edema, hemorrhage, or vasculitis	Presence of severe histologic plus immunopathologic findings

**Table 6:** Antibodies used for IHC reactions (VMS: Ventana Medical Systems).

Antibody (Clone)	Source	Manufacturer	Retrieval	Dilution
MMP2	VMS Inc.	Fine Biotech (Finetest)	High pH	1:50
MMP9	VMS Inc.	Fine Biotech (Finetest)	High pH	1:100
IL-1β	VMS Inc.	Fine Biotech (Finetest)	High pH	1:100

## Data Availability

No new data were created or analyzed in this study. Data are contained within the article.

## Abbreviations

The following abbreviations are used in this manuscript:

ACR	Acute Cellular Rejection
AMR	Antibody-Mediated Rejection
IL-1β	Interleukin-1 beta
MMP	Matrix Metalloproteinase
MMP2	Matrix Metalloproteinase 2
MMP9	Matrix Metalloproteinase 9
IHC	Immunohistochemistry
ECM	Extracellular Matrix
ISHLT	International Society for Heart and Lung Transplantation
DSA	Donor-Specific Antibodies
EGFR	Epidermal Growth Factor Receptor
PECAM1	Platelet and Endothelial Cell Adhesion Molecule 1 (CD31)
CD	Cluster of Differentiation
HE	Hematoxylin and Eosin
OR	Odds Ratio
CI	Confidence Interval
